# Synthesis of Conformationally Constrained d-Glu-*meso*-DAP Analogs as Innate Immune Agonists

**DOI:** 10.3390/molecules25225228

**Published:** 2020-11-10

**Authors:** Samo Guzelj, Žiga Jakopin

**Affiliations:** Department of Pharmaceutical Chemistry, Faculty of Pharmacy, University of Ljubljana, Aškerčeva 7, SI-1000 Ljubljana, Slovenia; samo.guzelj@ffa.uni-lj.si

**Keywords:** NOD1 agonist, constrained *meso*-DAP mimetics, NOD1 activation, innate immune agonist, bioisosteric replacement, rigidization

## Abstract

The dipeptide d-Glu-*mes*o-DAP (iE-DAP) is the minimal structural fragment capable of activating the innate immune receptor nucleotide-binding oligomerization domain protein (NOD1). The *meso*-diaminopimelic acid (*meso*-DAP) moiety is known to be very stringent in terms of the allowed structural modifications which still retain the NOD1 activity. The aim of our study was to further explore the chemical space around the *meso*-DAP portion and provide a deeper understanding of the structural features required for NOD1 agonism. In order to achieve the rigidization of the terminal amine functionality of *meso*-DAP, isoxazoline and pyridine heterocycles were introduced into its side-chain. Further, we incorporated the obtained *meso*-DAP mimetics into the structure of iE-DAP. Collectively, nine innovative iE-DAP derivatives additionally equipped with lauroyl or didodecyl moieties at the α-amino group of d-Glu have been prepared and examined for their NOD1 activating capacity. Overall, the results obtained indicate that constraining the terminal amino group of *meso*-DAP abrogates the compounds’ ability to activate NOD1, since only compound **6b** retained noteworthy NOD1 agonistic activity, and underpin the stringent nature of this amino acid with regard to the allowed structural modifications.

## 1. Introduction

Nucleotide-binding oligomerization domain protein (NOD)-like receptors (NLRs) constitute a family of innate immune receptors that mediate the response to bacterial peptidoglycan [1,2]. NOD1 is an extensively expressed prominent NLR representative which detects peptidoglycan fragments [3,4] featuring a dipeptide d-Glu-*mes*o-DAP (iE-DAP) motif in their structures [5,6,7,8]. Upon recognition of this sequence, NOD1 generates an immune response via the activation of nuclear factor κB (NF-κB) and mitogen-associated protein kinases [9,10,11], while it has also been implicated in other innate immunity mechanisms [12], such as caspase activation [13], apoptosis [14], and autophagy [15].

d-lactoyl-l-Ala-γ-d-Glu-*meso*-DAP-Gly (FK-156) and heptanoyl-γ-d-Glu-*meso*-DAP-d-Ala (FK-565) have been identified as some of the first NOD1 agonists [16,17]. These compounds have displayed significant therapeutic potential; besides reinforcing the innate and adaptive immune response, thereby increasing the host defence, they have also been shown to possess antitumor activity [18,19,20]. Recently, Agnihotri et al. conducted an extensive structure–activity relationship study, which revealed that by attaching lipophilic chains, in particular the lauroyl and didodecyl moieties, to the d-Glu α-amine functionality of iE-DAP, a several hundredfold more potent NOD1 agonistic activity could be achieved [21]. In line with these findings, our group designed iE-DAP derivatives decorated with lauroyl or didodecyl moieties at the amino group of the d-Glu residue. In addition, a double bond functionality was installed into the side-chain of the *meso*-diaminopimelic acid (*meso*-DAP) residue to increase the overall rigidity of these derivatives, which ultimately resulted in NOD1 agonists more potent than C12-iE-DAP [22]. In fact, the majority of the ensuing attempts to enhance its activity involved the introduction of lipophilic moieties into the d-glutamyl portion [23,24,25].

Based on the results of our previous work, we wanted to further explore the chemical space around the *meso*-DAP portion to provide a deeper understanding of the structural features required for NOD1 agonism. In order to achieve this objective, we explored possible replacements of this moiety and therefore turned our attention towards the *meso*-DAP mimetics reported in the literature to seek different options that provide optimization opportunities. The *meso*-DAP moiety is notorious for being very stringent as far as the allowed structural modifications, which still retain the NOD1 activity, are concerned. For example, Agnihotri et al. reported that no structural variations are allowed on its terminal amino group [21]. In spite of this, we investigated whether the rigidization of the terminal amine of the *meso*-DAP side-chain could bring about an increase in the NOD1 agonistic activity. To that end, we incorporated isoxazoline- and pyridine-based constrained *meso*-DAP mimetics into the structure of iE-DAP. In addition, lauroyl or didodecyl moieties were attached to the α-amino group of the d-Glu residue previously determined to be optimal. Collectively, nine innovative iE-DAP derivatives have been prepared as potential NOD1 agonists. The HEK-Blue NOD1 cell line served as a model for studying the ability of these compounds to activate NF-κB through NOD1 activation.

## 2. Results and Discussion

### 2.1. Design and Chemistry

**Figure 1:**
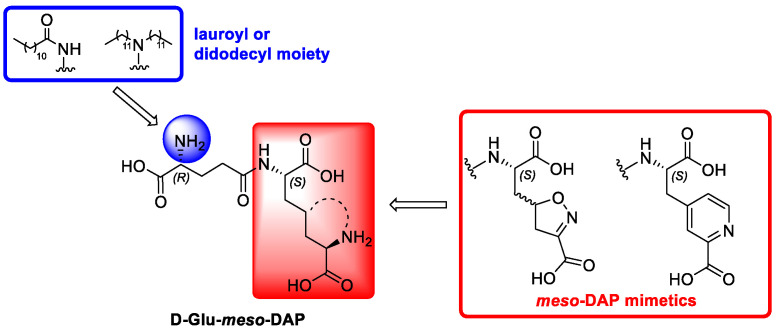
Design of conformationally constrained d-Glu-*mes*o-DAP analogs carrying *meso*-diaminopimelic acid (*meso*-DAP) mimetics.

Since diaminopimelic acid is featured in biosynthetic pathways and a number of biologically active compounds, it has received considerable attention. Due to the flexibility of its side-chain, *meso*-DAP is capable of assuming a number of spatial conformations. Previously, we have shown that installing a double bond functionality into the side chain can result in an increase in the NOD1 activating capacity of molecules [22]. As a continuation of our previous efforts, we postulated that constraining the terminal amino group of *meso*-DAP could also contribute to NOD1 agonistic activity. Several *meso*-DAP mimetics have been identified [26,27,28], including those carrying isoxazoline [29], pyridine [30], and aziridine [31] functionalities, whose structures are partially constrained, as well as *meso*-DAP bioisosteres, such as lanthionine [32], *meso*-oxa-DAP [33], and cystine [34], all of which still retain and mimic the key structural features of *meso*-DAP. We presumed that the rigidization of *meso*-DAP terminal amine by introducing either isoxazoline or pyridine moieties (see Figure 1) would best suit our objective and bring about an increase in the NOD1 agonistic activity.

**Scheme 1:**
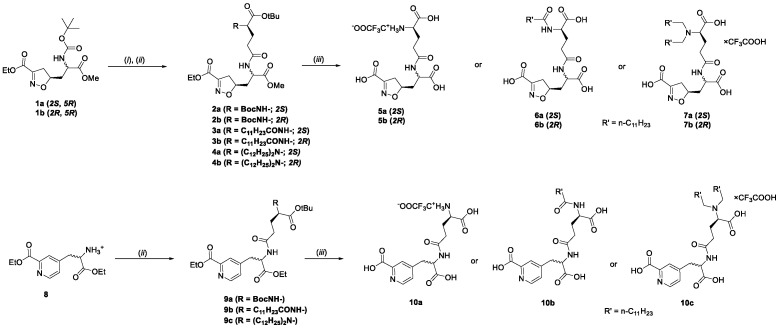
Synthesis of conformationally constrained analogs of iE-DAP. Reagents and conditions: (i) TFA/DCM (1:5), 0 °C then rt. (ii) Boc-d-Glu-O*t*Bu, *N*-lauroyl-d-Glu-O*t*Bu, or *N*,*N*-dilauryl-d-Glu-O*t*Bu, TBTU, diisopropylethylamine, DMAP/DCM. (iii) (a) 1M NaOH, MeOH; (b) TFA/DCM (1:1).

The synthetic strategy for preparing the conformationally constrained iE-DAP analogs is shown in Scheme 1. The starting diastereomeric isoxazoline mimetics of *meso*-DAP, **1a** ((2*S*,5*R*)-configuration) and **1b** ((2*R*,5*R*)-configuration), were synthesized according to the published procedure [29]. Next, *N*-Boc deprotection of **1a** and **1b** was achieved by acidolysis using a standard trifluoroacetic acid (TFA)-dichloromethane (DCM) (1:5) protocol [22]. In the following step, the resulting free amines were coupled with the commercially available Boc-d-Glu-O*t*Bu to obtain the fully protected d-Glu-*meso*-DAP derivatives **2a**–**b**. Alternatively, the free amines underwent in situ 2-(1*H*-benzotriazol-1-yl)-1,1,3,3-tetramethyluronium tetrafluoroborate (TBTU)-mediated coupling with lipophilic d-Glu derivatives *N*-lauroyl-d-Glu-O*t*Bu and *N*,*N*-dilauryl-d-Glu-O*t*Bu, thereby affording the fully protected d-Glu-*meso*-DAP mimetics **3a**–**b** and **4a**–**b**, respectively. Incidentally, *N*-lauroyl-d-Glu-O*t*Bu and *N*,*N*-dilauryl-d-Glu-O*t*Bu were synthesized as described previously [22]. Finally, the synthons **2a**–**b**, **3a**–**b,** and **4a**–**b** were subjected to a two-step deprotecting sequence entailing a classical alkaline hydrolysis utilizing 1M NaOH/MeOH and a subsequent acidolytic removal of the *N*-Boc and *O*-*t*Bu groups using a standard TFA-DCM (1:1) protocol to yield the isoxazoline-type d-Glu-*meso*-DAP mimetics **5a**–**b**, **6a**–**b,** and **7a**–**b**, respectively [29]. The synthesis of the pyridine-carrying d-Glu-*meso*-DAP mimetics is also illustrated in Scheme 1. The starting pyridine-based meso-DAP analog **8** carrying a free amine functionality was synthesized as previously described [30,35]. In the following step, TBTU-mediated coupling was employed to promote the acylation of the free amine with Boc-d-Glu-O*t*Bu and the lipophilic d-Glu derivatives *N*-lauroyl-d-Glu-O*t*Bu and *N*,*N*-dilauryl-d-Glu-O*t*Bu, thus furnishing the corresponding dipeptides **9a**, **9b,** and **9c**, respectively. Further, the deprotection of dipeptides **9a**–**c** with 1M NaOH/MeOH and a subsequent acidolysis yielded the desired pyridine-type d-Glu-*meso*-DAP mimetics **10a**, **10b,** and **10c**.

### 2.2. Biological Characterization

**Figure 2:**
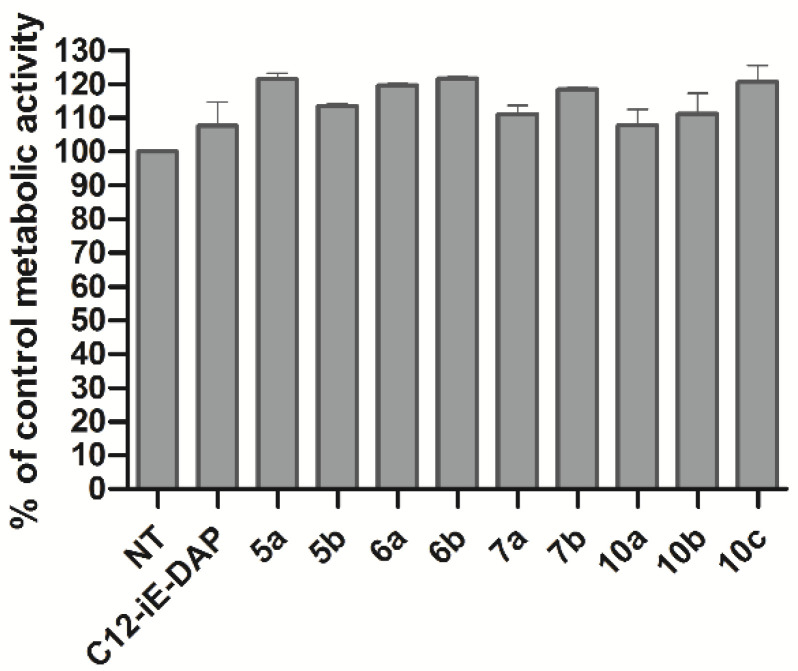
Proliferation rates of NOD1-specific HEK-Blue cells following 20 h of incubation with C12-iE-DAP (100 nM) and compounds **5a**–**b**, **6a**–**b**, **7a**–**b**, **10a**–**c** (10 μM). The rates, expressed as means of duplicates ± S.E.M. of two independent experiments, are shown relative to that of the control (NT).

Using the MTS metabolic activity assay, the proliferation rates of HEK-Blue NOD1 cells were evaluated in the presence of C12-iE-DAP and the novel constrained iE-DAP analogs to check for potential cytotoxicity. Cells were treated for 20 h with the compound of interest at concentrations of up to 10 μM. A comparison of the resulting metabolic activities with those of the untreated control showed that the compounds were well tolerated by HEK-Blue NOD1 cells, since none of their residual metabolic activities fell below 90% at the maximum concentration tested (Figure 2).

**Figure 3:**
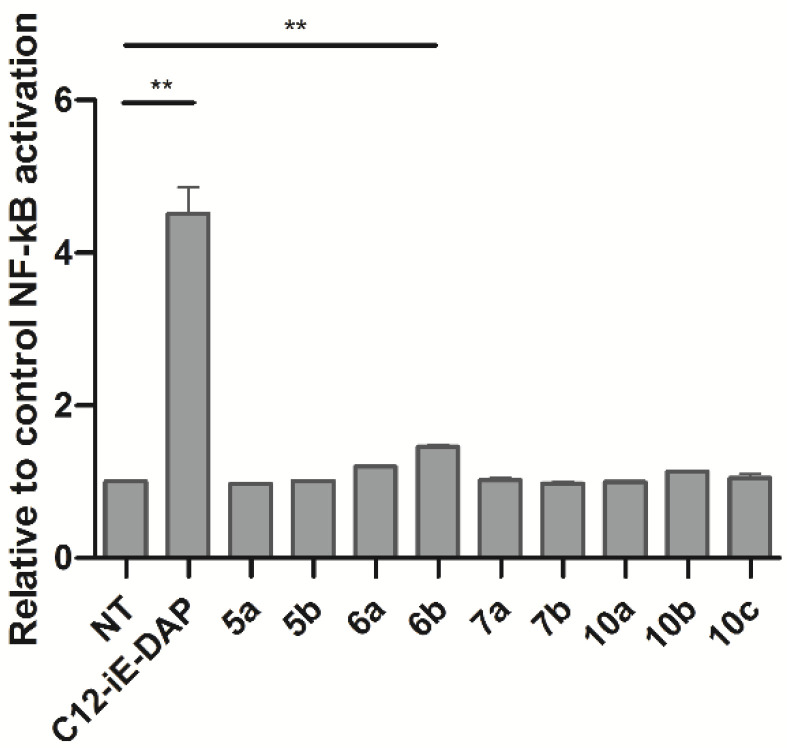
Effects of conformationally constrained lipophilic analogs of γ-d-glutamyl-*meso*-diaminopimelic acid **5a**–**b**, **6a**–**b**, **7a**–**b**, and **10a**–**c** on the NF-κB transcriptional activity. Secreted embryonic alkaline phosphatase (SEAP) activity was measured in HEK-Blue NOD1 cells after incubation for 20 h with C12-iE-DAP (100 nM) and synthesized compounds (10 µM). Columns represent means of duplicates of two independent experiments. Error bars indicate ± S.E.M.; ** *p* < 0.01 vs. untreated cells (ctrl).

The synthesized compounds and C12-iE-DAP were then examined for potential NOD1-activating capacity using the standard HEK-Blue NOD1 assay. HEK-Blue NOD1 cells stably express the human NOD1 gene and an NF-κB-inducible secreted embryonic alkaline phosphatase (SEAP) reporter gene. The recognition of a NOD1 agonist by its cognate receptor triggers a signaling cascade leading to the activation of NF-κB and the production of SEAP. HEK-Blue NOD1 cells were incubated for 20 h with C12-iE-DAP (100 nM) and compounds **5a**–**b**, **6a**–**b**, **7a**–**b**, and **10a**–**c** at concentrations of 10 μM. As expected, the positive control, C12-iE-DAP, increased the NF-κB transcriptional activity (4.51-fold) relative to that of untreated cells, while only a modest effect on this activity has been observed with the synthesized compounds, thus highlighting their lack of noteworthy NOD1-activating capacity (Figure 3). A compound that carries in its structure an *N*-lauroyl moiety attached to the d-Glu amino functionality and a (2*R*)-configuration was the best performing NOD1 agonist of the series (1.46-fold activation), while its diastereomer **6a** with a (2*S*)-configuration exhibited a weaker and insignificant NOD1 agonistic activity (1.20-fold activation). Interestingly, their close structural analogs **7a** and **7b**, in which the d-Glu amine groups are decorated with didodecyl moieties, were devoid of any activity. Moreover, the pyridine-based analog **10b**, which incorporates the lipophilic *N*-lauroyl moiety, exhibited only insignificant NOD1 activation (1.14-fold). Importantly, the obtained results show that constraining the terminal amine functionality of *meso*-DAP moiety abrogates the compounds’ ability to activate NOD1. Similarly, Vijayrajratnam et al. have reported that the amidation of the terminal carboxylic acid results in a loss of NOD1 agonistic activity [36]. In summation, our research further underlines the stringent nature of this amino acid with regard to structural modifications.

## 3. Materials and Methods

### 3.1. General Information

Chemicals were obtained from Acros, Aldrich Chemical Co., Molekula and Fluka, and used without further purification. C12-iE-DAP (a synthetic NOD1 agonist) was obtained from InvivoGen, Inc. (San Diego, CA, USA). Analytical TLC was performed on Merck 60 F254 silica gel plates (0.25 mm) using visualization with ultraviolet light and ninhydrin. Column chromatography was carried out on silica gel 60 (particle size 240–400 mesh). Melting points were determined on a Reichert hot stage microscope and are uncorrected. ^1^H- and ^13^C-NMR spectra were recorded at 400 and 100 MHz, respectively, on a Bruker AVANCE III spectrometer (Bruker, MA, USA) in DMSO-d_6_, CDCl_3_, or MeOD solution with TMS as the internal standard. Spectra were assigned using gradient COSY and HSQC experiments. IR spectra were recorded on a Perkin-Elmer 1600 FT-IR spectrometer (PerkinElmer, MA, USA). Microanalyses were performed on a 240 C Perkin-Elmer CHN analyzer (PerkinElmer, MA, USA). Mass spectra were obtained using a VG-Analytical Autospec Q mass spectrometer (Waters, Manchester, UK). HPLC analyses were performed on an Agilent Technologies HP 1100 instrument with G1365B UV-VIS detector (Agilent, CA, USA) (215, 220 or 254 nm), using a Luna C18 column (4.6 × 150 mm) at flow rate 1 mL/min. The eluent was a mixture of 0.1% TFA in water (A) and acetonitrile (B), with a gradient of 30% B to 80% B from 0 to 30 min and 80% B to 90% B from 30 to 33 min. The purity of all the pharmacologically investigated compounds was >95%. Stock solutions of chemicals were prepared in DMSO before use and the final concentration of DMSO never exceeded 0.2%.

### 3.2. General Procedures

#### 3.2.1. General Procedure for the Acidolytic Cleavage of Boc Protecting Groups and the Subsequent TBTU-Mediated Coupling

To an ice-chilled stirred mixture of trifluoroacetic acid and dichloromethane (1/5, 5 mL), Boc- protected compound (0.2 mmol) was added and the mixture was allowed to warm to room temperature. After 3 h, the reaction was completed and the solvent was evaporated in vacuo. The residue was washed three times with diethyl ether, giving sufficiently pure free amine, which was dissolved in dichloromethane (2 mL). In a parallel reaction, to a stirred solution of corresponding carboxylic acid derivative (0.2 mmol) in dry dichloromethane (10 mL), diisopropylethylamine (0.20 mmol), TBTU (0.22 mmol), and a catalytic amount of DMAP were added. After stirring for 45 min, the solution of free amine in dichloromethane was added at 0 °C, the mixture was allowed to warm to room temperature, and stirring then continued for 48 h. Upon completion, the reaction mixture was diluted with dichloromethane (30 mL) and then washed with 1M of HCl (2 × 20 mL), water (20 mL), saturated NaHCO_3_ solution (2 × 20 mL), and water (20 mL) and then dried over anhydrous Na_2_SO_4_. The solvent was concentrated in vacuo and the residue was purified by flash silica gel column chromatography (gradient elution; starting eluent: chloroform/methanol 20:1 *v*/*v*) to afford compounds **2a**–**b**, **3a**–**b**, **4a**–**b,** and **9a**–**c**.

#### 3.2.2. Final Cleavage of Methyl, Ethyl and *Tert*-Butyl Esters

To a solution of ester (0.075 mmol) in MeOH (2 mL) was added 1M of NaOH (6 eq; 0.45 mL) and the mixture was stirred for 1 h at room temperature. Upon the completion of the reaction, which was monitored by TLC, the reaction mixture was acidified with 2M of HCl to pH ~ 4–5 and then extracted with ethyl acetate (4 × 10 mL). The organic phase was dried over anhydrous Na_2_SO_4_ and evaporated in vacuo. The residue was then dissolved in an ice-chilled stirred mixture of trifluoroacetic acid and dichloromethane (1/1, 2 mL) and the mixture was allowed to warm to room temperature. After 24 h, the reaction was completed and the solvent was evaporated in vacuo. The residue was washed three times with diethyl ether, giving sufficiently pure compounds **5a**–**b**, **6a**–**b**, **7a**–**b,** and **10a**–**c**.

### 3.3. Characterization of Compounds

Ethyl (5*R*)-5-[(2*S*)-2-({(4*R*)-5-(*tert*-butoxy)-4-[(*tert*-butoxycarbonyl)amino]-5-oxopentanoyl}amino)-3-methoxy-3-oxopropyl]-4,5-dihydro-3-isoxazolecarboxylate (**2a**). Colourless oil, yield: 70 mg (66%); ^1^H-NMR (CDCl_3_, 400 MHz): δ = 1.38 (t, 3H, *J* = 7.2 Hz, CH_3_CH_2_), 1.46 (s, 9H, 3 × CH_3_), 1.48 (s, 9H, 3 × CH_3_), 1.81–1.91 (m, 1H, CH_2A_-β-Glu), 2.19–2.27 (m, 3H, CH_2B_-β-Glu, CHCH_2_CH), 2.35–2.39 (m, 2H, CH_2_-γ-Glu), 2.90–2.97 (m, 1H, C_q_-CH_2A_), 3.35–3.43 (m, 1H, C_q_-CH_2B_), 3.80 (s, 3H, CH_3_O), 4.35 (q, 2H, *J* = 7.2 Hz, CH_3_CH_2_), 4.64–4.69 (m, 1H, CH-Glu), 4.91–4.98 (m, 1H, CH-O), 5.25 (dd, 1H, *J* = 8.0 Hz, *J* = 16.4 Hz, NH), 6.80 (d, 1H, *J* = 6.4 Hz, NH), 7.06 (d, 1H, *J* = 6.8 Hz, NH) ppm. MS (ESI): *m*/*z* (%) = 530.3 (M + H)^+^. IR (KBr): *ν* = 2974, 1711, 1510, 1363, 1248, 1150 cm**^−^**^1^. HRMS Calcd for C_24_H_40_N_3_O_10_ *m/z*: 530.2714 (M + H)^+^, found 530.2706. [α]_D_^20^ = +18.8° (*c* 0.15, CH_2_Cl_2_).

Ethyl (5*R*)-5-[(2*R*)-2-({(4*R*)-5-(*tert*-butoxy)-4-[(*tert*-butoxycarbonyl)amino]-5-oxopentanoyl}amino)-3-methoxy-3-oxopropyl]-4,5-dihydro-3-isoxazolecarboxylate (**2b**). Colourless oil, yield: 75 mg (71%); ^1^H-NMR (CDCl_3_, 400 MHz): δ = 1.38 (t, 3H, *J* = 7.2 Hz, CH_3_CH_2_), 1.46 (s, 9H, 3 × CH_3_), 1.48 (s, 9H, 3 × CH_3_), 1.85–1.90 (m, 1H, CH_2A_-β-Glu), 2.09–2.28 (m, 3H, CH_2B_-β-Glu, CHCH_2_CH), 2.35 (t, 2H, *J* = 7.2 Hz, CH_2_-γ-Glu), 2.88–2.95 (m, 1H, C_q_-CH_2A_), 3.32–3.39 (m, 1H, C_q_-CH_2B_), 3.78 (s, 3H, CH_3_O), 4.36 (q, 2H, *J* = 7.2 Hz, CH_3_CH_2_), 4.70–4.75 (m, 1H, CH-Glu), 4.91–4.98 (m, 1H, CH-O), 5.27 (dd, 1H, *J* = 8.8 Hz, *J* = 20.0 Hz, NH), 6.86 (d, 1H, *J* = 7.2 Hz, NH), 7.20 (d, 1H, *J* = 7.2 Hz, NH) ppm. MS (ESI): *m/z* (%) = 530.3 (M + H)^+^. IR (KBr): *ν* = 3420, 2359, 2090, 1642 cm**^−^**^1^. HRMS Calcd for C_24_H_40_N_3_O_10_ *m*/*z*: 530.2714 (M + H)^+^, found 530.2718. [α]_D_^20^ = +15.0° (*c* 0.20, CH_2_Cl_2_).

Ethyl (*R*)-5-((*S*)-2-((*R*)-5-(*tert*-butoxy)-4-dodecanamido-5-oxopentanamido)-3-methoxy-3-oxopropyl)-4,5-dihydroisoxazole-3-carboxylate (**3a**). Colourless oil, yield: 98 mg (80%); ^1^H-NMR (CDCl_3_, 400 MHz): δ = 0.90 (t, 3H, *J* = 6.8 Hz, CH_3_(CH_2_)_10_CO), 1.18–1.21 (m, 18H, CH_3_(CH_2_)_9_CH_2_CO), 1.39 (t, 3H, *J* = 6.8 Hz, CH_3_CH_2_O), 1.47 (s, 9H, O*t*Bu), 1.83–1.93 (m, 1H, CH_2A_-β-Glu), 2.17–2.30 (m, 5H, CH_2B_-β-Glu, CH_2_CO, isoxazoline-CH_2_-CH), 2.31–2.40 (m, 2H, CH_2_CO), 2.94 (dd, 1H, *J* = 9.6 Hz, *J* = 8.0 Hz, isoxazoline-H_4a_), 3,40 (m, 1H, isoxazoline-H_4b_), 3.80 (s, 3H, COOCH_3_), 4.37 (q, 2H, *J* = 7.2 Hz, CH_3_CH_2_O), 4.59–4.71 (m, 2H, 2 × CH), 4.91–4.99 (m, 1H, isoxazoline-H_5_), 6.28–6.35 (m, 2H, NH) ppm. ^13^C-NMR (CDCl_3_, 100 MHz): δ = 14.1, 22.7, 25.7, 28.0, 29.1, 29.3, 29.4, 29.5, 29.6, 29.7, 31.9, 32.4, 32.5, 36.7, 37.2, 37.4, 39.1, 39.2, 49.9, 52.0, 52.8, 62.2, 80.5, 82.7, 151.5, 160.5, 171.3, 171.8, 172.2, 172.5, 173.8 ppm. MS (ESI): *m*/*z* (%) = 612.4 (M+H)^+^. IR (ATR): *ν* = 587, 650, 748, 850, 930, 1016, 1130, 1158, 1254, 1348, 1369, 1439, 1531, 1589, 1646, 1723, 2853, 2924, 3331 cm**^−^**^1^. HRMS Calcd for C_31_H_54_N_3_O_9_ *m*/*z*: 612.3860 (M + H)^+^, found 612.3863. [α]_D_^20^ = −10° (*c* 0.08, CH_2_Cl_2_).

Ethyl (*R*)-5-((*R*)-2-((*R*)-5-(*tert*-butoxy)-4-dodecanamido-5-oxopentanamido)-3-methoxy-3-oxopropyl)-4,5-dihydroisoxazole-3-carboxylate (3b). Colourless oil, yield: 70 mg (57%); ^1^H-NMR (CDCl_3_, 400 MHz): δ = 0.88 (t, 3H, *J* = 7.2 Hz, CH_3_(CH_2_)_10_CO), 1.26–1.30 (m, 16H, CH_3_(CH_2_)_8_CH_2_CO), 1.37 (t, 3H, *J* = 7.2 Hz, CH_3_CH_2_O), 1.47 (s, 9H, O*t*Bu), 1.67 (m, 2H, CH_3_(CH_2_)_8_CH_2_CH_2_), 1.80–1.92 (m, 1H, CH_2A_-β-Glu), 2.12–2.33 (m, 7H, CH_2B_-β-Glu, 2 × CH_2_CO, isoxazoline-CH_2_-CH), 2.89–2.93 (m, 1H, isoxazoline-H_4a_), 3,34 (dd, 1H, *J* = 11.2 Hz, *J* = 6.4 Hz, isoxazoline-H_4b_), 3.77 (s, 3H, COOCH_3_), 4.35 (q, 2H, *J* = 7.2 Hz, CH_3_CH_2_O), 4.54–4.65 and 4.67–4.80 (m, 2H, 2 × CH), 4.89–4.97 (m, 1H, isoxazoline-H_5_), 6.30–6.36 (m, 1H, NH), 7.03 and 7.49 (2d, 1H, *J* = 7.6 Hz, NH) ppm. ^13^C-NMR (CDCl_3_, 100 MHz): δ = 14.1, 22.7, 25.6, 25.7, 28.0, 29.1, 29.4, 29.5, 29.6, 29.7, 31.9, 32.5, 36.7, 37.0, 37.1, 39.1, 39.2, 50.0, 50.2, 52.0, 52.1, 52.6, 52.7, 62.2, 80.6, 80.7, 82.7, 151.6, 160.5, 171.3, 171.8, 172.2, 172.5, 173.7, 173.9 ppm. MS (ESI): *m*/*z* (%) = 612.4 (M + H)^+^. IR (ATR): *ν* = 574, 689, 748, 845, 930, 1016, 1125, 1160, 1255, 1346, 1370, 1436, 1536, 1591, 1647, 1724, 2853, 2923, 3313 cm**^−^**^1^. HRMS Calcd for C_31_H_54_N_3_O_9_ *m*/*z*: 612.3860 (M + H)^+^, found 612.3863. [α]_D_^20^ = +5.7° (*c* 0.07, CH_2_Cl_2_).

Ethyl (*R*)-5-((*S*)-2-((*R*)-5-(*tert*-butoxy)-4-(didodecylamino)-5-oxopentanamido)-3-methoxy-3-oxopropyl)-4,5-dihydroisoxazole-3-carboxylate (**4a**). Colourless oil, yield: 72 mg (47%); ^1^H-NMR (CDCl_3_, 400 MHz): δ = 0.89 (t, 9H, *J* = 7.2 Hz, 2 × CH_3_CH_2_-), 1.24–1.27 (m, 40H, 2 × CH_3_(CH_2_)_10_CH_2_), 1.38 (t, 3H, *J* = 7.2 Hz, CH_3_CH_2_O), 1.45 (s, 9H, O*t*Bu), 1.56–1.65 (m, 2H, CH_2_), 1.81–2.08 (m, 2H, CH_2_CH), 2.15–2.97 (m, 8H, CH_2_CO, 2 × CH_2_N, isoxazoline-CH_2_-CH), 3.24 (dd, 1H, *J* = 6.4 Hz, *J* = 2.4 Hz, isoxazoline-H_4a_), 3.38 (dd, 1H, *J* = 10.8 Hz, *J* = 6.8 Hz, isoxazoline-H_4b_), 3.79 (s, 3H, COOCH_3_), 4.05–4.08 (m, 1H, CH), 4.36 (q, 2H, *J* = 7.2 Hz, CH_3_CH_2_O), 4.63–4.68 (m, 1H, CH), 4.84–4.93 (m, 1H, isoxazoline-H_5_), 6.40–6.44 (m, 1H, NH) ppm. ^13^C-NMR (CDCl_3_, 100 MHz): δ = 14.1, 22.7, 23.1, 23.6, 25.2, 25.3, 26.9, 27.0, 27.2, 27.4, 28.0, 28.2, 28.3, 29.0, 29.4, 29.6, 29.7, 31.9, 32.9, 33.0, 35.3, 37.5, 37.6, 39.2, 41.9, 49.8, 51.2, 51.6, 52.8, 60.6, 62.2, 62.8, 63.0, 64.6, 80.2, 80.3, 80.8, 82.2, 82.5, 151.5, 160.5, 169.2, 171.3, 171.9, 172.3, 172.9, 175.2 ppm. MS (ESI): *m*/*z* (%) = 766.6 (M + H)^+^. IR (ATR): *ν* = 722, 747, 849, 929, 1019, 1147, 1251, 1368, 1464, 1526, 1676, 1721, 2853, 2923 cm^−1^. HRMS Calcd for C_43_H_80_N_3_O_8_ *m*/*z*: 766.5945 (M + H)^+^, found 766.5944. [α]_D_^20^ = +41.5° (*c* 0.07, CH_2_Cl_2_).

Ethyl (*R*)-5-((*R*)-2-((*R*)-5-(*tert*-butoxy)-4-(didodecylamino)-5-oxopentanamido)-3-methoxy-3-oxopropyl)-4,5-dihydroisoxazole-3-carboxylate (**4b**). Colourless oil, yield: 90 mg (59%); ^1^H-NMR (CDCl_3_, 400 MHz): δ = 0.88 (t, 6H, *J* = 6.4 Hz, 2 × CH_3_CH_2_-), 1.24–1.26 (m, 40H, 2 × CH_3_(CH_2_)_10_CH_2_), 1.36 (t, 3H, *J* = 7.2 Hz, CH_3_CH_2_O), 1.45 (s, 9H, O*t*Bu), 1.53–1.60 (m, 2H, CH_2_), 1.77–1.89 (m, 1H, CH_2A_-β-Glu), 1.91–2.67 (m, 5H, CH_2B_-β-Glu, CH_2_CO, isoxazoline-CH_2_-CH), 2.72–2.95 (m, 4H, 2×CH_2_N), 3.22–3.25 (m, 1H, isoxazoline-H_4a_), 3.33 (dd, 1H, *J* = 10.4 Hz, *J* = 7.2 Hz, isoxazoline-H_4b_), 3.77 (s, 3H, COOCH_3_), 4.05 (d, 1H, *J* = 8.8 Hz, CH(NH)), 4.36 (q, 2H, *J* = 6.8 Hz, CH_3_CH_2_O), 4.69–4.78 (m, 1H, CH), 4.84–4.94 (m, 1H, isoxazoline-H_5_), 6.38–6.39 (m, 1H, NH) ppm. ^13^C-NMR (CDCl_3_, 100 MHz): δ = 14.1, 22.7, 23.1, 23.5, 25.2, 26.7, 27.0, 27.2, 27.4, 28.0, 28.2, 28.3, 29.1, 29.4, 29.6, 29.7, 29.8, 31.9, 32.9, 35.6, 37.5, 39.2, 41.9, 50.0, 51.2, 51.7, 52.7, 60.7, 62.2, 62.9, 64.8, 80.8, 82.2, 82.7, 151.6, 160.5, 169.0, 171.3, 171.8, 172.3, 172.8, 174.8, 175.3 ppm. MS (ESI): *m*/*z* (%) = 766.6 (M + H)^+^. IR (ATR): *ν* = 731, 847, 929, 1020, 1147, 1251, 1367, 1461, 1533, 1589, 1678, 1722, 2853, 2923 cm^−1^. HRMS Calcd for C_43_H_80_N_3_O_8_ *m*/*z*: 766.5945 (M + H)^+^, found 766.5966. [α]_D_^20^ = +27.1° (*c* 0.07, CH_2_Cl_2_).

(1*R*)-1-carboxy-4-({(1*S*)-1-carboxy-2-[(5*R*)-3-carboxy-4,5-dihydro-5-isoxazolyl]ethyl}amino)-4-oxo-1-butanaminium 2,2,2-trifluoroacetate (**5a**). Colourless oil, yield: 33 mg (74%); ^1^H-NMR (DMSO-d_6_, 400 MHz): δ = 1.95–2.11 (m, 4H, CH_2_-β-Glu, CHCH_2_CH), 2.26–2.42 (m, 2H, CH_2_-γ-Glu), 2.86–2.92 (m, 1H, C_q_-CH_2A_), 3.21–3.29 (m, 1H, C_q_-CH_2B_), 3.93–3.95 (m, 1H, CH), 4.29–4.35 (m, 1H, CH), 4.78–4.84 (m, 1H, CH-O), 8.27 (s, 3H, NH_3_^+^), 8.42 (d, 1H, *J* = 8.0 Hz, NH), 13.44 (br s, 3H, COOH) ppm. ^13^C-NMR (DMSO-d_6_, 100 MHz): δ = 23.2, 25.2, 26.2, 26.4, 30.0, 30.7, 30.9, 31.8, 37.9, 47.2, 49.0, 52.3, 52.4, 63.5, 72.7, 118.0, 118.8, 157.9, 170.5, 171.4, 173.3, 174.4 ppm. MS (ESI): *m*/*z* (%) = 332.1 (M + H)^+^. IR (KBr): *ν* = 3421, 2359, 1669, 1437, 1202, 1141, 802, 724 cm^−1^. HRMS Calcd for C_12_H_18_N_3_O_8_ *m*/*z*: 332.1094 (M + H)^+^, found 332.1097. Anal. Calcd for C_12_H_18_N_3_O_8_ × 0.4 CF_3_COOH × 0.3 CH_3_CH_2_OCH_2_CH_3_ (%): C, 37.45; H, 4.20; N, 8.19. Found: C, 37.29; H, 4.20; N, 8.18. [α]_D_^20^ = −7.3° (*c* 0.28, MeOH).

(1*R*)-1-carboxy-4-({(1*R*)-1-carboxy-2-[(5*R*)-3-carboxy-4,5-dihydro-5-isoxazolyl]ethyl}amino)-4-oxo-1-butanaminium 2,2,2-trifluoroacetate (**5b**). Colourless oil, yield: 38 mg (85%); ^1^H-NMR (DMSO-d_6_, 400 MHz): δ = 1.81–1.88 (m, 1H, CH_2A_-β-Glu), 1.95–2.11 (m, 3H, CH_2B_-β-Glu, CHCH_2_CH), 2.27–2.41 (m, 2H, CH_2_-γ-Glu), 2.86–2.92 (m, 1H, C_q_-CH_2A_), 3.23–3.30 (m, 1H, C_q_-CH_2B_), 3.90–4.00 (m, 1H, CH), 4.29–4.35 (m, 1H, CH), 4.72–4.79 (m, 1H, CH-O), 8.29 (s, 3H, NH_3_^+^), 8.38 (d, 1H, J = 8.0 Hz, NH) ppm. ^13^C-NMR (DMSO-d_6_, 100 MHz): δ = 14.4, 18.4, 26.8, 27.0, 27.1, 32.1, 32.7, 37.5, 39.6, 51.1, 53.5, 58.4, 63.1, 63.9, 64.9, 81.7, 119.0, 153.4, 162.3, 170.2, 170.7, 171.5, 173.5, 174.5, 174.6 ppm. MS (ESI): *m/z* (%) = 332.1 (M + H)^+^. IR (KBr): *ν* = 2932, 1667, 1432, 1198, 1142, 800, 723 cm^−1^. HRMS Calcd for C_12_H_18_N_3_O_8_ *m/z*: 332.1094 (M + H)^+^, found 332.1090. Anal. Calcd for C_12_H_18_N_3_O_8_ × 0.3 CF_3_COOH × 0.15 CH_3_CH_2_OCH_2_CH_3_ (%): C, 37.21; H, 4.07; N, 8.56. Found: C, 36.80; H, 4.45; N, 8.18. [α]_D_^20^ = −2.2° (*c* 0.22, MeOH).

(*R*)-5-((*S*)-2-carboxy-2-((*R*)-4-carboxy-4-dodecanamidobutanamido)ethyl)-4,5-dihydroisoxazole-3-carboxylic acid (**6a**). Light brown oil, yield: 28 mg (73%); ^1^H-NMR (MeOD, 400 MHz): δ = 0.91 (t, 3H, *J* = 6.8 Hz, CH_3_(CH_2_)_10_CO), 1.30–1.33 (m, 16H, CH_3_(CH_2_)_8_), 1.64 (t, 2H, *J* = 6.8 Hz, CH_3_(CH_2_)_8_CH_2_), 1.92–2.05 (m, 1H, CH_2A_-β-Glu), 2.11–2.32 (m, 7H, CH_2B_-β-Glu, isoxazoline-CH_2_, 2 × CH_2_CO), 2.97 (dd, 1H, *J* = 8.0 Hz, *J* = 9.6 Hz, isoxazoline-H_4a_), 3.32–3.41 (m, 1H, isoxazoline-H_4b_), 4.40–4.47 (m, 1H, CH), 4.55–4.58 (m, 1H, CH), 4.91–5.00 (m, 1H, isoxazoline-H_5_) ppm. ^13^C-NMR (MeOD, 100 MHz): δ = 14.5, 15.5, 23.8, 27.0, 28.3, 28.5, 30.4, 30.5, 30.7, 30.8, 33.1, 36.9, 37.9, 39.8, 50.9, 51.0, 51.1, 53.1, 53.2, 67.0, 82.7, 111.6, 114.6, 117.4, 117.5, 118.6, 127.4, 128.4, 135.3, 153.7, 163.2, 174.4, 174.8, 174.9, 175.0, 176.6 ppm. MS (ESI): *m*/*z* (%) = 512.3 (M − H)^−^. IR (ATR): *ν* = 720, 930, 1023, 1085, 1205, 1377, 1439, 1540, 1626, 1721, 2361, 2854, 2923 cm^−1^. HRMS Calcd for C_24_H_38_N_3_O_9_ *m*/*z*: 512.2608 (M − H)^−^, found 512.2609. [α]_D_^20^ = −3.6° (*c* 0.06, CH_2_Cl_2_). HPLC (215 nm): 97.5%, t_r_ = 18.16 min.

(*R*)-5-((*R*)-2-carboxy-2-((*R*)-4-carboxy-4-dodecanamidobutanamido)ethyl)-4,5-dihydroisoxazole-3-carboxylic acid (**6b**). Brown amorphous solid, yield: 24 mg (63%); mp 85-87 °C; ^1^H-NMR (MeOD, 400 MHz): δ = 0.94 (t, 3H, *J* = 7.2 Hz, CH_3_(CH_2_)_10_), 1.31–1.34 (m, 16H, CH_3_(CH_2_)_8_CH_2_CO), 1.64 (t, 2H, *J* = 6.8 Hz, CH_3_(CH_2_)_8_CH_2_CO), 1.96–2.03 (m, 1H, CH_2A_-β-Glu), 2.15–2.31 (m, 5H, CH_2B_-β-Glu, 2 × CH_2_CO), 2.39–2.43 (m, 2H, isoxazoline-CH_2_), 2.92–2.99 (m, 1H, isoxazoline-H_4a_), 3.36–3.40 (m, 1H, isoxazoline-H_4b_), 4.40–4.45 (m, 1H, CH), 4.58–4.63 (m, 1H, CH), 4.86–4.95 (m, 1H, isoxazoline-H_5_) ppm. ^13^C-NMR (MeOD, 100 MHz): δ = 14.5, 23.8, 26.9, 28.3, 28.5, 30.4, 30.5, 30.7, 30.8, 33.1, 33.2, 36.9, 37.7, 37.9, 39.7, 50.9, 53.2, 53.3, 81.8, 153.9, 163.2, 174.7, 175.0, 175.1, 176.6 ppm. MS (ESI): *m*/*z* (%) = 512.3 (M-H)^−^. IR (ATR): *ν* = 590, 719, 800, 929, 1134, 1236, 1434, 1539, 1620, 1719, 2853, 2923 cm^−1^. HRMS Calcd for C_24_H_38_N_3_O_9_ *m*/*z*: 512.2608 (M−H)^−^, found 512.2602. [α]_D_^20^ = +10° (*c* 0.05, MeOH). HPLC (215 nm): 95.3%, t_r_ = 18.04 min.

*N*-((*R*)-1-carboxy-4-(((*S*)-1-carboxy-2-((*R*)-3-carboxy-4,5-dihydroisoxazol-5-yl)ethyl)amino)-4-oxobutyl)-*N*-dodecyldodecan-1-aminium 2,2,2-trifluoroacetate (**7a**). Brown amorphous solid, yield: 56 mg (95%); mp 93–96 °C; ^1^H-NMR (MeOD, 400 MHz): δ = 0.91 (t, 6H, *J* = 6.8 Hz, 2 × CH_3_(CH_2_)_11_), 1.30–1.38 (m, 36H, 2 × CH_3_(CH_2_)_9_CH_2_), 1.77–1.79 (m, 4H, 2 × CH_3_(CH_2_)_9_CH_2_CH_2_), 2.13–2.40 (m, 4H, CH_2_-β-Glu, CH_2_CH_2_CO), 2.66–2.68 (m, 2H, isoxazoline-CH_2_), 2.93–3.01 (m, 1H, isoxazoline-H_4a_), 3.15–3.35 (m, 4H, 2 × CH_2_N), 3.36–3.41 (m, 1H, isoxazoline-H_4b_), 4.17–4.23 (m, 1H, CH), 4.57–4.66 (m, 1H, CH) ppm. ^13^C-NMR (MeOD, 100 MHz): δ = 15.5, 23.8, 25.8, 27.6, 30.2, 30.5, 30.7, 30.8, 33.1, 37.8, 40.0, 51.3, 53.1, 53.9, 54.0, 158.8, 159.2, 162.5, 163.2, 163.4, 163.5, 170.8, 170.9, 173.2, 174.3, 174.4, 174.5 ppm. MS (ESI): *m/z* (%) = 668.5 (M + H)^+^. IR (ATR): *ν* = 599, 723, 801, 842, 1135, 1182, 1439, 1674, 2855, 2925 cm^−1^. HRMS Calcd for C_36_H_66_N_3_O_8_ *m*/*z*: 668.4850 (M + H)^+^, found 668.4863. [α]_D_^20^ = +2.2° (*c* 0.09, MeOH). HPLC (215 nm): 95.3%, t_r_ = 32.12 min.

*N*-((*R*)-1-carboxy-4-(((*R*)-1-carboxy-2-((*R*)-3-carboxy-4,5-dihydroisoxazol-5-yl)ethyl)amino)-4-oxobutyl)-*N*-dodecyldodecan-1-aminium 2,2,2-trifluoroacetate (**7b**). Brown amorphous solid, yield: 52 mg (88%); mp 91–93 °C; ^1^H-NMR (MeOD, 400 MHz): δ = 0.91 (t, 6H, *J* = 6.8 Hz, 2 × CH_3_(CH_2_)_11_), 1.31–1.39 (m, 36H, 2 × CH_3_(CH_2_)_9_CH_2_), 1.75–1.85 (m, 4H, 2 × CH_3_(CH_2_)_9_CH_2_CH_2_), 1.96–2.50 (m, 4H, CH_2_-β-Glu, CH_2_CH_2_CO), 2.62–2.78 (m, 2H, isoxazoline-CH_2_), 2.93–3.02 (m, 1H, isoxazoline-H_4a_), 3.15–3.37 (m, 4H, 2 × CH_2_N), 3.36–3.42 (m, 1H, isoxazoline-H_4b_), 4.20–4.22 (m, 1H, CH), 4.64–4.67 (m, 1H, CH) ppm. ^13^C-NMR (MeOD, 100 MHz): δ = 14.5, 23.8, 24.1, 25.8, 27.6, 28.0, 30.2, 30.4, 30.5, 30.7, 30.8, 33.1, 37.6, 39.7, 43.1, 50.9, 54.0, 61.2, 81.8, 113.8, 116.7, 119.6, 154.1, 162.7, 163.1, 163.3, 174.6, 175.3, 178.2 ppm. MS (ESI): *m*/*z* (%) = 668.5 (M + H)^+^. IR (ATR): *ν* = 599, 723, 801, 841, 943, 1135, 1182, 1434, 1672, 2855, 2925 cm^−1^. HRMS Calcd for C_36_H_66_N_3_O_8_ *m*/*z*: 668.4850 (M + H)^+^, found 668.4857. [α]_D_^20^ = +8.0° (*c* 0.05, MeOH). HPLC (215 nm): 100%, t_r_ = 32.00 min.

Ethyl 4-[2-({(4*R*)-5-(*tert*-butoxy)-4-[(*tert*-butoxycarbonyl)amino]-5-oxopentanoyl}amino)-3-ethoxy-3-oxopropyl]-2-pyridinecarboxylate (**9a**). Colourless oil, yield: 47 mg (43%); ^1^H-NMR (DMSO-d_6_, 400 MHz): δ = 1.12–1.20 (m, 6H, 2 × CH_3_CH_2_), 1.38 (s, 18H, 6×CH_3_), 1.57–1.69 and 1.73–1.83 (2m, 1H each, CH_2_-β-Glu), 2.10–2.16 (m, 2H, CH_2_-γ-Glu), 2.97–3.04 (m, 1H, Py-CH_2A_), 3.13–3.18 (m, 1H, Py-CH_2B_), 3.71–3.78 (m, 1H, CH), 4.00–4.10 (m, 4H, 2 × CH_3_CH_2_), 4.51–4.57 (m, 1H, CH), 7.08–7.12 (m, 1H, NH), 7.49–7.52 (m, 1H, Py-H), 7.93 (s, 1H, Py-H), 8.37 (dd, 1H, *J* = 3.2 Hz, *J* = 8.0 Hz, Py-H), 8.60 (d, 1H, *J* = 5.2 Hz, NH) ppm. MS (ESI): *m*/*z* (%) = 552.3 (M + H)^+^. IR (KBr): *ν* = 3325, 2977, 2361, 1720, 1517, 1368, 1301, 1206, 1153, 1023 cm^−1^. HRMS Calcd for C_27_H_42_N_3_O_9_ *m/z*: 552.2921 (M + H)^+^, found 552.2911. [α]_D_^20^ = −8.0° (*c* 0.10, CH_2_Cl_2_).

Ethyl 4-(2-((*R*)-5-(*tert*-butoxy)-4-dodecanamido-5-oxopentanamido)-3-ethoxy-3-oxopropyl)picolinate (**9b**). Brown oil, yield: 43 mg (27%); ^1^H-NMR (CDCl_3_, 400 MHz): 0.88 (t, 3H, *J* = 7.2 Hz, CH_3_(CH_2_)_10_), 1.24-1.32 (m, 24H, CH_3_(CH_2_)_8_CH_2_, 2 × CH_3_CH_2_O), 1.44 (s, 9H, O*t*Bu), 1.63–1.65 (m, 2H, CH_2_CH_2_CONH), 1.77–1.87 (m, 1H, CH_2A_-β-Glu), 2.07–2.38 (m, 5H, CH_2B_-β-Glu, 2 × CH_2_CO) 3.13–3.20 and 3.25–3.32 (2m, 1H each, Pyr-CH_2_-), 4.20 (q, 2H, *J* = 7.2 Hz, CH_3_CH_2_O),4.31–4.35 (m, 1H, CH), 4.44–4.51 (m, 2H, CH_3_CH_2_O), 4.84–4.89 (m, 1H, NHCHCOOEt), 6.36 (dd, 1H, *J* = 15.2 Hz, *J* = 8.0 Hz, NH), 7.03 and 7.52 (2d, 1H (2 × 0,5), *J* = 7.6 Hz, NH), 7.36 (d, 1H, *J* = 4.8 Hz, Py-H), 7.99 (d, 1H, *J* = 12.4 Hz, Py-H), 8.67 (d, 1H, *J* = 4.8 Hz, Py-H) ppm. ^13^C-NMR (CDCl_3_, 100 MHz): δ = 14.1, 14.3, 22.7, 25.6, 27.9, 29.3, 29.5, 29.6, 29.9, 31.9, 32.5, 36.7, 37.1, 38.6. 51.9, 52.0, 52.9, 61.8, 61.9, 62.0, 82.8. 126.0, 126.1, 127.7, 147.1, 147.2, 148.3, 149.9, 165.1, 165.2, 170.8, 171.0, 171.2, 171.4, 172.0, 172.3, 173.7, 173.9 ppm. MS (ESI): *m*/*z* (%) = 634.4 (M + H)^+^. IR (ATR): *ν* = 589, 655, 686, 730, 786, 851, 928, 995, 1029, 1064, 1128, 1157, 1209, 1246, 1300, 1367, 1458, 1538, 1605, 1644, 1727, 2854, 2923, 3301 cm^−1^. HRMS Calcd for C_34_H_56_N_3_O_8_ *m/z*: 634.4067 (M + H)^+^, found 634.4078. [α]_D_^20^ = −5.0° (*c* 0.06, CH_2_Cl_2_).

Ethyl 4-(2-((*R*)-5-(*tert*-butoxy)-4-(didodecylamino)-5-oxopentanamido)-3-ethoxy-3-oxopropyl)picolinate (**9c**). Brown oil, yield: 74 mg (47%); ^1^H-NMR (CDCl_3_, 400 MHz): *δ* = 0.90 (t, 6H, *J* = 6.8 Hz, 2 × CH_3_(CH_2_)_11_), 1.23–1.27 (m, 40H, 2 × CH_2_(CH_2_)_10_CH_3_), 1.30 (t, 6H, *J* = 7.2 Hz, 2 × CH_3_CH_2_O), 1.45 (s, 9H, O*t*Bu), 1.80–2.03 (m, 2H CH_2_-β-Glu), 2.22–2.39 (m, 2H, CH_2_-γ-Glu), 2.43–2.49 (m, 2H, CH_2_N), 2.60–2.62 (m, 2H, -CH_2_N), 3.18 (m, 2H, CH_2_), 3.18–3.30 (m, 1H, CH), 4.17–4.26 (m, 2H, CH_3_CH_2_O), 4.50 (q, 2H, CH_3_CH_2_O), 4.87–4.93 (m, 1H, NHCHCOOEt), 6.16 (s, 1H, Py-H), 7.30 (m, 1H, NHCO), 7.94 (s, 1H, Py-H), 8.68 (d, 1H, *J* = 4.8 Hz, Py-H) ppm. ^13^C-NMR (CDCl_3_, 100 MHz): δ = 14.1, 14.4, 22.7, 25.2, 25.3, 27.4, 28.3, 29.1, 29.4, 29.7, 31.9, 32.8, 33.0, 37.4, 37.5. 51.2, 52.5, 62.0, 62.8, 63.0, 80.8, 126.0, 127.7, 146.8, 148.4, 149.9, 165.1, 170.8, 170.9, 172.3, 172.5 ppm. MS (ESI): *m*/*z* (%) = 788.6 (M + H)^+^. IR (ATR): *ν* = 645, 731, 784, 850, 918, 1025, 1147, 1204, 1251, 1300, 1367, 1464, 1529, 1602, 1676, 1722, 2853, 2923 cm**^−^**^1^. HRMS Calcd for C_46_H_82_N_3_O_7_ m/z: 788.6153 (M + H)^+^, found 788.6141. [α]_D_^20^ = +41.8° (*c* 0.06, CH_2_Cl_2_).

(1*R*)-1-carboxy-4-{[1-carboxy-2-(2-carboxy-4-pyridinyl)ethyl]amino}-4-oxo-1-butanaminium 2,2,2-trifluoroacetate (**10a**). White amorphous powder, yield: 31 mg (92%); m.p. 198–200 °C. ^1^H-NMR (DMSO-d_6_, 400 MHz): δ = 1.89–1.93 (m, 2H, CH_2_-β-Glu), 2.15–2.35 (m, 2H, CH_2_-γ-Glu), 2.95–3.01 (m, 1H, Py-CH_2A_), 3.18–3.23 (m, 1H, Py-CH_2B_), 3.81–3.94 (m, 1H, CH), 4.51–4.57 (m, 1H, CH), 7.51 (dd, 1H, *J* = 0.8 Hz, *J* = 4.8 Hz, Py-H), 7.96 (d, 1H, *J* = 0.8 Hz, Py-H), 8.27 (s, 3H, NH_3_^+^), 8.45 (dd, 1H, *J* = 4.0 Hz, *J* = 8.0 Hz, Py-H), 8.60 (d, 1H, *J* = 5.2 Hz, NH) ppm. ^13^C-NMR (DMSO-d_6_, 100 MHz): δ = 14.1, 25.8, 25.9, 30.3, 30.4, 35.7, 48.5, 51.4, 52.3, 115.6, 118.6, 125.4, 127.6, 148.2, 148.4, 149.1, 157.6, 157.9, 158.2, 158.5, 166.1, 170.7, 170.9, 172.4 ppm. MS (ESI): *m*/*z* (%) = 340.1 (M + H)^+^. IR (KBr): *ν* = 3298, 2885, 1653, 1534, 1194, 799 cm**^−^**^1^. HRMS Calcd for C_14_H_18_N_3_O_7_ *m*/*z*: 340.1145 (M + H)^+^, found 340.1131. Anal. Calcd for C_14_H_17_N_3_O_7_ × 0.8 CF_3_COOH (%): C, 43.52; H, 4.17; N, 9.76. Found: C, 43.11; H, 4.06; N, 9.54. [α]_D_^20^ = −19.2° (*c* 0.07, MeOH).

4-(2-carboxy-2-((*R*)-4-carboxy-4-dodecanamidobutanamido)ethyl)picolinic acid (**10b**). Brown oil, yield: 22 mg (83%); ^1^H-NMR (MeOD, 400 MHz): 0.92 (t, 3H, *J* = 7.2 Hz, CH_3_(CH_2_)_10_), 1.20–1.40(m, 16H, CH_3_(CH_2_)_8_CH_2_CH_2_), 1.62 (m, 2H, CH_3_(CH_2_)_8_CH_2_CH_2_), 1.80–1.93 and 2.04–2.14 (2m, 2H, CH_2_-β-Glu), 2.23–2.30 (m, 4H, 2 × CH_2_CO), 3.16–3.23 and 3.45–3.58 (2m, 1H, Py-CH_2_), 4.27–4.34 (m, 1H, CH), 4.82–4.88 (m, 1H, CH), 7.70–7.75 (m, 1H, Pyr-H), 8.19–8.22 (m, 1H, Py-H), 8.66–8.70 (m, 1H, Py-H) ppm. ^13^C-NMR (MeOD, 100 MHz): *δ* = 14.5, 23.8, 26.9, 30.3, 30.5, 30.7, 30.8, 33.1, 36.8, 36.9, 37.8, 37.9, 53.1, 53.5, 53.7, 114.6, 117.5, 127.7, 129.9, 147.5, 147.6, 149.6, 152.3, 165.8, 173.6, 174.8, 176.5 ppm. MS (ESI): *m*/*z* (%) = 520.3 (M − H)**^−^**. IR (ATR): *ν* = 593, 664, 720, 786, 977, 1085, 1134, 1186, 1308, 1443, 1539, 1643, 1730, 2853, 2923 cm**^−^**^1^. HRMS Calcd for C_26_H_38_N_3_O_8_ m/z: 520.2659 (M − H)**^−^**, found 520.2645. [α]_D_^20^ = +14.5° (*c* 0.06, MeOH). HPLC (254 nm): 48.6%, t_r_ = 14.84 min.

*N*-((1*R*)-1-carboxy-4-((1-carboxy-2-(2-carboxypyridin-4-yl)ethyl)amino)-4-oxobutyl)-*N*-dodecyldodecan-1-aminium 2,2,2-trifluoroacetate (**10c**). Brown amorphous solid, yield: 56 mg (94%); mp 88–92 °C; ^1^H-NMR (MeOD, 400 MHz): δ = 0.92 (t, 6H, *J* = 7.2 Hz, 2 × CH_3_(CH_2_)_11_), 1.31–1.37 (m, 36H, 2 × CH_3_(CH_2_)_9_CH_2_), 1.69–1.73 (m, 4H, 2 × CH_3_(CH_2_)_9_CH_2_), 1.94–2.13 (m, 2H, CH_2_-β-Glu), 2.44–2.59 (m, 2H, CH_2_CH_2_CO), 3.04–3.22 (m, 4H, 2 × CH_2_N), 3.47–3.50 (m, 2H, Py-CH_2_), 3.71–3.76 (m, 1H, CH), 4.72–4.78 (m, 1H, CH), 7.56–7.59 (m, 1H, Py-H), 8.10 (d, 1H, *J* = 5.6 Hz, Py-H), 8.58–8.60 (m, 1H, Py-H) ppm. ^13^C-NMR (MeOD, 100 MHz): δ = 14.5, 23.7, 23.8, 25.5, 27.6, 30.2, 30.5, 30.7, 30.8, 33.1, 53.1, 53.4, 54.5, 67.1, 113.9, 116.8, 119.7, 127.3, 129.6, 150.5, 155.8, 162.9, 163.3, 166.1, 174.7 ppm. MS (ESI): *m*/*z* (%) = 676.5 (M + H)^+^. IR (ATR): *ν* = 574, 722, 801, 839, 1130, 1179, 1204, 1325, 1405, 1674, 2855, 2924 cm^−1^. HRMS Calcd for C_38_H_66_N_3_O_7_ *m*/*z*: 676.4901 (M + H)^+^, found 676.4899. [α]_D_^20^ = +7.1° (*c* 0.07, MeOH). HPLC (254 nm): 92.8%, t_r_ = 12.52 min.

### 3.4. Cytotoxicity Assay 

HEK-Blue NOD1 cells (Invivogen, San Diego, CA, USA) were cultured in accordance with the manufacturer’s instructions. HEK-Blue NOD1 cells (4 × 10^5^ cells/mL; 40,000/well) were treated with the appropriate amounts of compounds or with the corresponding vehicle (control cells), then seeded in duplicate in 96-well plates. After 20 h, the metabolic activity was assessed using the CellTiter 96^®^ Aqueous One Solution Cell Proliferation Assay (Promega, Madison, WI, USA), in accordance with the manufacturer’s instructions. The results are expressed as the means of duplicates ± S.E.M. of two independent experiments.

### 3.5. Measurement of NF-κB Transcriptional Activity (Quanti-Blue Assay) 

HEK-Blue NOD1 cells (Invivogen, San Diego, CA, USA) were cultured in accordance with the manufacturer’s instructions. HEK-Blue NOD1 cells were assayed for changes in the NF-κB transcriptional activity upon incubation (2.5 × 10^5^ cells/mL; 25,000/well) with C12-iE-DAP (100 nM) and synthesized compounds (10 µM) for 20 h. The secreted embryonic alkaline phosphatase (SEAP) activity was determined in the supernatant in accordance with the manufacturer’s instructions. Absorbance was measured on a BioTek Synergy microplate reader (BioTek Instruments, Inc., Winooski, VT, USA) at 640 nm. The results are expressed as the means of duplicates ± S.E.M. of two independent experiments.

### 3.6. Data Analysis and Statistics

All the experiments were performed at least two times, with average values expressed as means ± standard error of mean (SEM). Statistical analyses were performed using GraphPad Prism 6 (La Jolla, CA, USA). Statistical significance was determined with the unpaired *t*-test test. Differences were considered significant (*) for *P* < 0.05, highly significant (**) for *P* < 0.01, and extremely significant (***) for *P* < 0.001.

## 4. Conclusions

In conclusion, we have synthesized new constrained mimetics of the NOD1 agonist d-Glu-*meso*-DAP as potential innate immune agonists. The rigidization of the terminal amine group was achieved by introducing isoxazoline and pyridine heterocycles into the side chain of the *meso*-DAP residue. In addition, the α-amino group of the d-Glu residue of iE-DAP was either *N*-acylated or *N*,*N*-dialkylated, rendering the molecule considerably more lipophilic. The results obtained have demonstrated that the isoxazoline- and pyridine-carrying analogs do not properly recapitulate the key structural features of *meso*-DAP. Consequently, they exhibit only limited NOD1 agonistic activity. Nevertheless, our results offer additional insight into the chemical space of d-Glu-*meso*-DAP derivatives and underpin the stringent nature of *meso*-DAP with regard to the allowed structural modifications.

## Abbreviations

C12-iE-DAP	lauroyl-γ-d-glutamyl-*meso*-diaminopimelic acid
iE-DAP	γ-d-glutamyl-*meso*-diaminopimelic acid
*meso*-DAP	*meso*-diaminopimelic acid
NF-κB	nuclear factor κB
NLR	NOD-like receptor
NOD	nucleotide-binding oligomerization domain
SEAP	secreted embryonic alkaline phosphatase
TBTU	2-(1*H*-benzotriazol-1-yl)-1,1,3,3-tetramethyluronium tetrafluoroborate
TFA	trifluoroacetic acid
